# ﻿A new species of *Diplatys* (Insecta, Dermaptera, Diplatyidae) earwig from the Western Ghats of India

**DOI:** 10.3897/zookeys.1088.79416

**Published:** 2022-03-07

**Authors:** Chikkabidare M. Karthik, Yoshitaka Kamimura, Chicknayakanahalli M. Kalleshwaraswamy

**Affiliations:** 1 Department of Entomology, College of Agriculture, Keladi Shivappa Nayaka University of Agricultural and Horticultural Sciences, Navile, Shivamogga – 577204, Karnataka, India Keladi Shivappa Nayaka University of Agricultural and Horticultural Sciences Shivamogga India; 2 Department of Biology, Keio University, 4-1-1 Hiyoshi, Yokohama 223-8521, Japan Keio University Yokohama Japan

**Keywords:** *Diplatyssahyadriensis* sp. nov., Karnataka, Sri Lanka, sugarcane

## Abstract

To explore diversity of earwigs (Dermaptera) in different agricultural ecosystems of South India, an extensive taxonomic survey was conducted in 2020 during which an undescribed species of *Diplatys* was collected. Twenty-one species of the genus *Diplatys* (Diplatyidae, Diplatyinae) have been reported to date from India, of which six species are known from Karnataka, South India. Based on a male specimen collected from a sugarcane field in Karnataka, a new species, *Diplatyssahyadriensis***sp. nov.**, is described as the twenty-second species of this genus from India. A revised key to the males of *Diplatys* species from India and Sri Lanka is provided. This new record adds to the known species diversity in the Sahyadri Ranges of the Western Ghats in Shivamogga District, Karnataka, part of the Southern Plateau and Hills agro-climatic region of India.

## ﻿Introduction

Earwigs are a moderately diversified group of insects which comprise approximately 1,900 species distributed mainly in tropical and subtropical parts of the world (Hopkins et al. 2018). The previous taxonomic study by Srivastava (2013) reported 284 species from India. Although the taxonomy is unstable and unsettled, the genus *Diplatys* (Diplatyidae), in a broad sense, is species rich with approximately 70 described species from the Ethiopian and Oriental regions (Hopkins et al. 2018). According to the system of Srivastava (1988, 2013), 25 species of *Diplatys* have been reported from India and Sri Lanka, of which four species are endemic to Sri Lanka (Srivastava 1988, 1989, 2013; Table 1); clearly these two countries represent the centre of *Diplatys* diversity.

**Table 1.** Distribution and subgeneric classification of *Diplatys* species recorded from India and Sri Lanka.

Species	Subgenus by Steinmann (1986b)	Distribution						Sri Lanka
		Highland (Himalayas & N.E. mountains)	Humid Subtropical	Tropical Wet and Dry	Arid	Semiarid	Tropical Wet	
*D.adjacens* Hincks, 1955	(*Syndiplatys*)		Uttarakhand^1^, Madhya Pradesh^1^	Madhya Pradesh^1^				
*D.anamaliensis* Srivastava, 1970	(*Syndiplatys*)					Tamil Nadu^1^		
*D.brindlei* Steinmann, 1974	(*Neodiplatys*)	Central Himalaya^3^	West Bengal^1,4^					
*D.carinatus* Srivastava, 1988	Not assigned			Karnataka^1^			Karnataka^1^	
*D.carli* Srivastava, 1988	Not assigned			Tamil Nadu^1^				
*D.chopardi* Hincks, 1955	(*Neodiplatys*)			Karnataka^1^ Tamil Nadu^1^		Tamil Nadu^1^		
*D.chowdhuryi* Srivastava, 1989	Not assigned			Odisha^2^				
*D.coelebs* Hincks, 1955	(*Syndiplatys*)			Maharashtra^1^, Tamil Nadu^1^			Karnataka^1^, Kerala^1^	
*D.confusus* Hincks, 1955	(*Syndiplatys*)			Tamil Nadu^1^		Tamil Nadu^1^		
*D.devlensis* Srivastava, 1974	(*Hypodiplatys*)			Tamil Nadu^1^				
*D.dolens* Hincks, 1957	(*Neodiplatys*)			Maharashtra^1^				
*D.ernesti* Burr, 1910	(*Syndiplatys*)							Uva Province^1^, Central Province^1^
*D.excidens* Hincks, 1954	(*Neodiplatys*)			Karnataka^1^				
*D.fletcheri* Burr, 1910	(*Hypodiplatys*)		Madhya Pradesh^1^	Tamil Nadu^1^		Tamil Nadu^1^		Uva Province^1^
*D.greeni* Burr, 1904	(*Syndiplatys*)							Central Province^1^, Sabaragamuwa Province^1^
*D.jawalagriensis* Kapoor, Bharadwaj & Banerjee, 1971	(*Diplatys*)			Karnataka^1^, Tamil Nadu^1^		Tamil Nadu^1^		
*D.lefroyi* Burr, 1910	(*Neodiplatys*)			Karnataka^1^				
*D.menoni* Kapoor & Bharadwaj, 1968	(*Diplatys*)			Maharashtra^1^				
*D.nathani* Hincks, 1960	(*Diplatys*)		Madhya Pradesh^1^					
*D.nilgiriensis* Hincks, 1955	(*Syndiplatys*)			Tamil Nadu^1^				
*D.papovi* Bey-Bienko, 1959	(*Neodiplatys*)		Meghalaya^1^					
*D.propinquus* Hincks, 1955	(*Syndiplatys*)							Central Province^1^
*D.sahyadriensis* **sp. nov.**	Not assigned			Karnataka^3^				
*D.santoshi* Srivastava, 1975	(*Syndiplatys*)							Central Province^1^
*D.sinuatus* Hincks, 1955	(*Syndiplatys*)	Himachal Pradesh^1,^ North Western Himalayas^3^	Bihar^1^, Odisha^1^, West Bengal^4^	West Bengal^4^				
*D.tikaderi* Srivastava, 1988	Not assigned			Odisha^1^				

1. Srivastava (1988), 2. Srivastava (1989), 3. Deepak and Ghosh (2018), 4. Srivastava (1993a)

To explore the diversity of earwigs in different agricultural ecosystems of South India, we conducted an extensive taxonomic survey in agricultural and horticultural crop fields. Here we report a new species, *D.sahyadriensis* sp. nov., based on a male specimen collected from a sugarcane ecosystem. The possible relationships of the new species with other *Diplatys* recorded from India and Sri Lanka and the diversity of this genus in this region are also discussed.

## ﻿Materials and methods

The specimen was collected by hand from a sugarcane field in Shivamogga District, Karnataka, India, and preserved in 70% ethanol. For the morphological identification, the specimen was examined under a Stemi 508 stereozoom microscope (Carl Zeiss Microscopy GmbH, Jena, Germany). Photographs of the habitus and external body parts were taken under an M205C stereozoom microscope attached with a DFC450 camera (Leica, Wetzlar, Germany). The male genitalia were removed by gently lifting the penultimate abdominal sternite, pulling out from the genital chamber with forceps, and cutting at the site of attachment to the ejaculatory ducts. The genitalia were processed by submerson in 5% KOH for two days for clearing tissues and mounted on a glass slide with glycerol. Photographs of dissected genitalia were taken an M205C stereozoom microscope attached with a DFC450 camera. Based on the photographs, the genitalia were illustrated using Adobe Illustrator CS6. The specimen, with voucher number UAHSE19K, is deposited in the Insect Systematics and Vector Biology Laboratory, Department of Entomology, College of Agriculture, Keladi Shivappa Nayaka University of Agricultural and Horticultural Sciences, Shivamogga. The terminology of Kamimura (2014) was adopted to describe male genital structures.

## ﻿Taxonomy

### ﻿Order Dermaptera de Geer, 1773

**Infraorder Protodermaptera Zacher, 1910**

**Family Diplatyidae Verhoeff, 1902**

**Subfamily Diplatyinae Verhoeff, 1902**

#### Genus *Diplatys* Audinet-Serville, 1831

##### ﻿ Diplatys sahyadriensis

Taxon classificationAnimaliaDermapteraDiplatyidae

Karthik, Kamimura & Kalleshwaraswamy sp. nov.

0F2675BE-CF0D-5C69-988C-46BA9D01B25D

http://zoobank.org/3E7D2B09-09B4-437B-93B1-280FEBD35207

**Figure 1.**
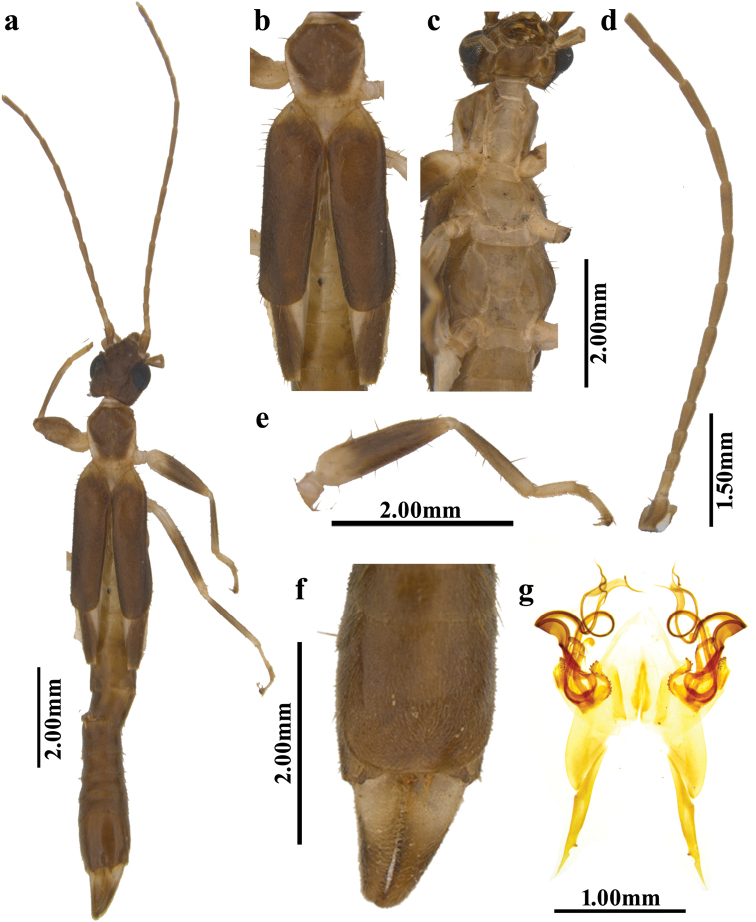
*Diplatyssahyadriensis* sp. nov. (male holotype) **a** habitus **b** pronotum, tegmina and wings **c** thoracic sterna **d** left antenna **e** right foreleg **f** penultimate sternite and forceps **g** genitalia.

**Figure 2.**
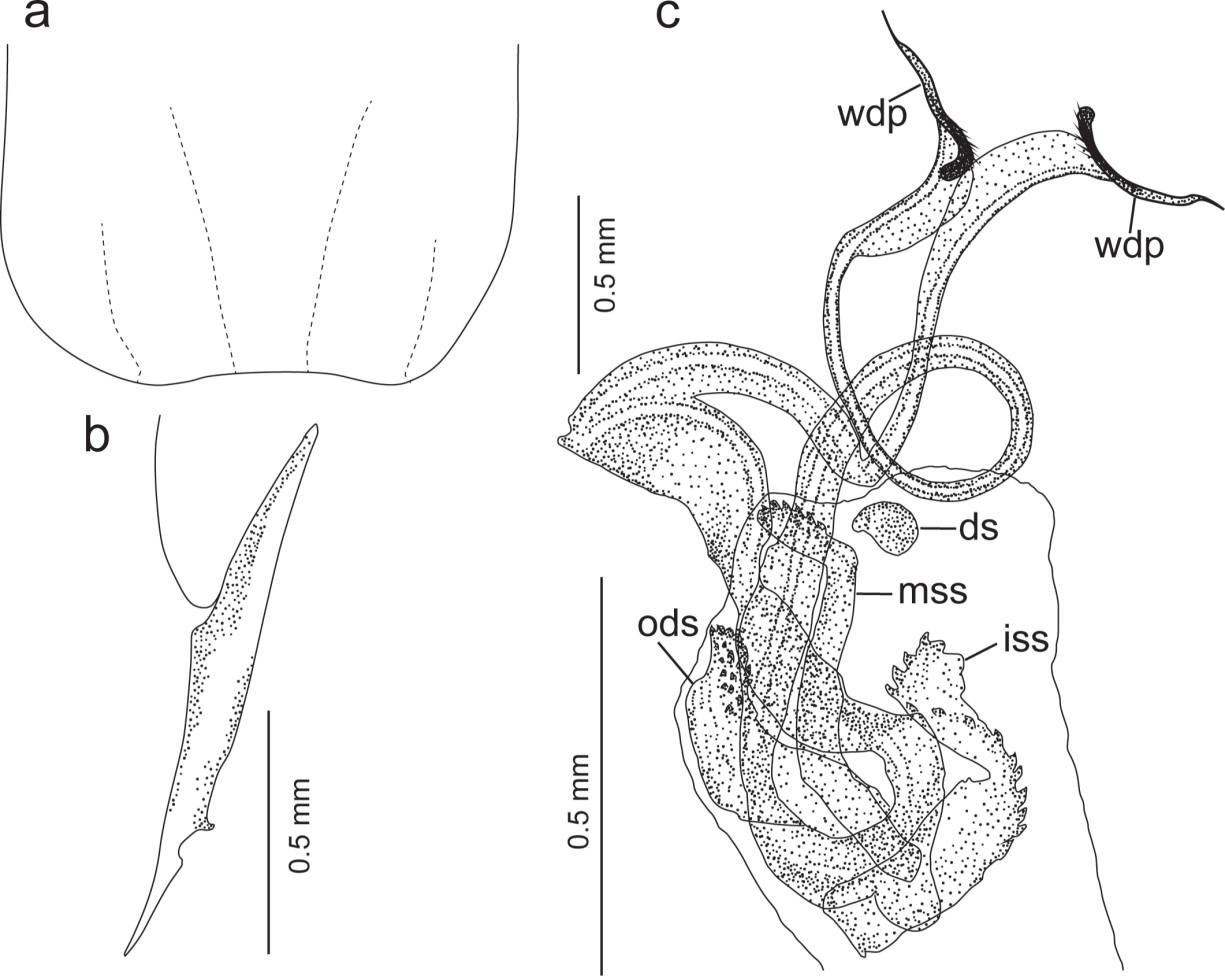
*Diplatyssahyadriensis* sp. nov. (male holotype) **a** posterior half of penultimate sternite **b** left paramere **c** left penis (in repose), virga with whip-like distal processes (wdp), and associated sclerites (ds, disc-shaped sclerite; iss, inner serrated sclerite; mss, mid serrated sclerite; ods, outer denticulated sclerite).

Figs 1 , 2 , Table 2

###### Material examined.

***Holotype*** (♂), India: Karnataka, Hosanagara-Shivamogga Road, Galigekola, 13°59'52.854"N, 75°22'42.576"E, 6.xi.2020, C.M. Karthik leg., *ex.* sugarcane.

###### Diagnosis.

Male has simple forceps, and is easily discriminated from that of other *Diplatys* species by the unique morphology of the virga: paired portion with developed flanges and a whip-like process at each tip. Flanged virgae have been reported in this genus only for *D.jawalagiriensis* Kapoor, Bharadwaj & Banerjee, 1971. However, almost no unpaired part is present at the base of each virga in *D.jawalagiriensis*, with no associated large sclerites in the penis lobe (vs short but conspicuous unpaired part and characteristic associated sclerites are present in *D.sahyadriensis* sp. nov.).

###### Description.

**Male** (holotype: Figs 1, 2). Measurements of body parts are shown in Table 2. Body generally dark brown. 2^nd^ antennal segment and beyond light brown. Coxa, trochanter, basal third of femur, distal half of tibia, tarsi, base of tegmina, wings (excluding fustis), lateral and posterior margins of pronotum, and base of forceps whitish brown (Fig. 1a–f). Abdomen and forceps densely pubescent (Fig. 1a, f).

**Table 2.** Measurements (mm) of the male holotype of *Diplatyssahyadriensis* sp. nov.

Length	Measurement (mm)
Body without forceps	11.12
Head	1.55
Pronotum	1.22
Tegmen	3.00
Forceps	1.30
**Width**	
Head	1.57
Pronotum	1.22
Tegmen	1.00
Ultimate tergite	1.25

Head (Fig. 1a) slightly wider than long, widest in eye region; frons tumid but weakly depressed at apex; occiput strongly and widely depressed; transverse and median sutures visible but not conspicuous; posterior margin strongly emarginated in middle. Eyes prominent, distinctly longer than the post-ocular length. Antennae (Fig. 1a, d) 17 segments or more (in holotype 15 left segments and 17 right segments remain), 1^st^ expanded apically, slightly shorter than the combined length of 2^nd^ to 4^th^; 2^nd^ minute, shorter than width; 3^rd^ long and slender; 4^th^ slightly shorter than 3^rd^; 5^th^ onwards segments gradually increasing in length and thinning up to 13^th^ and 14^th^. Pronotum (Fig. 1b) longer than broad, narrowed posteriorly, anteriorly convex, sides almost straight, hind margin subrotundate, median sulcus distinct, prozona tumid and well differentiated from flat metazona. Tegmina (Fig. 1b) well developed, humeral angles weak, costal margin straight, posterior margin obliquely convex, axillary angles weak, showing a broad triangular scutellum. Wings (Fig. 1b) well developed.

Prosternum (Fig. 1c) elongate, with a constriction at the point of attachment of forelegs. Mesosternum (Fig. 1c) broader than prosternum, more or less rounded, deeply constricted at the point of attachment of the midlegs, truncated posteriorly. Metasternum (Fig. 1c) hexagonal, constricted at point of attachment of hindlegs, emarginate posteriorly.

Abdomen (Fig. 1a) long, cylindrical, greatly enlarging from 7^th^ tergite onwards. Penultimate sternite (Figs 1f, 2a), relatively long, posterior margin weakly emarginated at middle. Ultimate tergite (Fig. 1a) transverse with two small, bifid, undulate depressions. Forceps (Fig. 1a, f) about as long as the ultimate tergite, trigonal with ridge only present in basal two-thirds, branches tapering apically with pointed tip and without curving.

Parameres (= external parameres; Figs 1g, 2b) with an internal tooth at apical one-quarter and a small, deep concavity distal to it. Penis lobes (Figs 1g, 2c) slightly shorter than the parameres, each with a denticulated sclerite (= outer denticulated sclerite; ods), two differently shaped, serrated sclerites (mid serrated sclerite [mss] and inner serrated sclerite [iss]), a disc-shaped sclerite (ds), and a bifurcated virga. Virga (Figs 1g, 2c) with very short, unpaired part, and long paired part. Each branch of paired part convoluted, slender, but with well-developed flange, and tips widened with a whip-like distal process (wdp).

**Female.** Unknown.

###### Etymology.

The specific epithet *sahyadriensis* refers to the type locality: the specimen was collected from the Sahyadri Ranges, which is a gateway to the Western Ghats of Karnataka.

###### Distribution.

Only known from Shivamogga, Karnataka, India.

###### Bioecology.

The male specimen (holotype) was collected from whorls of sugarcane *Saccharumofficinarum* L. (Angiospermae, Gramineae). The collection site is in a mixed area of sugarcane and paddy fields. Faecal pellets were seen on the leaves of sugarcane, suggesting that specimen had been in that place for some time, possibly taking advantage of this shaded spot.

### ﻿Key to *Diplatys* species known from India and Sri Lanka (males only)

(Adopted from Srivastava 1988; * = species known only from Sri Lanka)

**Table:** 

1	Virga much reduced, represented by dots; instead, a long chitinous rod projects from penis lobe (According to Srivastava 1989)	***Diplatyschowdhuryi* Srivastava, 1989**
–	Bifurcated virga present	**2**
2	Virga paired throughout its entire length	***Diplatysconfusus* Hincks, 1955; *Diplatyscoelebs* Hincks, 1955; *Diplatysjawalagiriensis* Kapoor, Bharadwaj & Banerjee, 1971; *Diplatysnathani* Hincks, 1960; *Diplatysdolens* Hincks, 1957; *Diplatystikaderi* Srivastava, 1988; *Diplatyslefroyi* Burr, 1910; or *Diplatysmenoni* Kapoor & Bharadwaj, 1968; see couplets 12–25 in the key by Srivastava (1988)**
–	Virga unpaired at base, sometimes forming a rounded vesicle or small protuberance; its length variable in relation to paired portion	**3**
3	Unpaired portion of virga about as long as or longer than the paired portion	*Diplatyspropinquus* Hincks, 1955*; *Diplatysernesti* Burr, 1910*; *Diplatysadjacens* Hincks, 1955; *Diplatyssinuatus* Hincks, 1955; *Diplatyssantoshi* Srivastava, 1975*; *Diplatysnilgiriensis* Hincks, 1955; or *Diplatyscarinatus* Srivastava, 1988; see couplets 37–48 in the key by Srivastava (1988)
–	Unpaired basal portion of virga very short to one-third of the paired portion	**4**
4	Parameres internally with a sharp tooth at about the middle, apical half narrow and points externally. Unpaired part of virga short but dilated, forming an oval vesicle	***Diplatysbrindlei* Steinmann, 1974**
–	Outer margin of parameres almost straight, apical half not obliquely pointing outward. Unpaired part of virga not forming a conspicuous oval-shaped vesicle	**5**
5	Posterior margin of penultimate sternite with a pair of conspicuous projections and emarginate between these projections	***Diplatyspopovi* Bey-Bienko, 1959**
–	Posterior margin of penultimate sternite almost truncate, emarginate, or sinuate, but without conspicuous projections	**6**
6	Posterior margin of penultimate sternite almost truncate, or only feebly undulate	**7**
–	Posterior margin of penultimate sternite emarginate at middle or bisinuate	**8**
7	Virga as long as parameres	***Diplatysdevlensis* Srivastava, 1974**
–	Virga longer than parameres, sometimes exceeding base of genitalia	***Diplatysfletcheri* Burr, 1910**
8	Posterior margin of penultimate sternite emarginate at middle or bisinuate. Virga very long, extending beyond base of genitalia	**9**
–	Penultimate sternite posteriorly emarginate at middle. Virga short or long, but not extending beyond base of genitalia	**10**
9	Penultimate sternite posteriorly bisinuate. Virga very long, extending beyond base of genitalia, paired part not flanged	***Diplatyschopardi* Hincks, 1955**
–	Penultimate sternite weakly emerged at middle posteriorly. Paired part of virga convoluted and laterally provided with flange, each apex with a whip-like process	***Diplatyssahyadriensis* Karthik, Kamimura & Kalleshwaraswamy, sp. nov.**
10	Virga apparently longer than penis lobe	***Diplatysexcidens* Hincks, 1954**
–	Virga shorter than or almost as long as penis lobe	**11**
11	Virga much shorter than 1/2 of penis lobe; paired part stout and swollen	***Diplatyscarli* Srivastava, 1988**
–	Virga longer than 1/2 of penis lobe; paired part slender	**12**
12	Inner pre-apical tooth of parameres strongly hooked	***Diplatysanamaliensis* Srivastava, 1970**
–	Inner pre-apical tooth of parameres normal, not strongly hooked	***Diplatysgreeni* Burr, 1904**

## ﻿Discussion

In the present study, we follow Srivastava’s (1988, 2013) classification of Diplatyinae, except for the treatment of *Haplodiplatys* Hincks, 1955, which is considered the sole genus of the family Haplodiplatyidae (Engel et al. 2017). Based mainly on the parameric characters, Steinmann (1974, 1986b) proposed to classify *Diplatys* sensu Hincks (1955) into four genera (*Diplatys*, *Schizodiplatys* Steinmann, 1974, *Lobodiplatys* Steinmann, 1974, and *Circodiplatys* Steinmann, 1986). Although Steinmann (1974) placed *Diplatysconradti* Burr, 1904 in *Lobodiplatys*, Zacher (1910) erected subgenus Paradiplatys Zacher, 1910, treating this species as its type. According to the Principle of Priority, Sakai (1982) resurrected the subgenus Paradiplatys as a full genus and synonymised *Lobodiplatys* with it. Engel and Haas (2007), who omitted to cite Sakai (1982), made the same proposal. This view was followed by Srivastava (1988, 2013) in classifying the Indian species of Diplatyinae into three genera: *Diplatys*, *Paradiplatys*, and *Nannopygia* Dohrn, 1863; Srivastava (1993b) considered *Nannopygia* a senior synonym of *Schizodiplatys*. Males of *Diplatys* possess a pair of elongate parameres with unarmed external margins (vs armed with a single movable epimerite in *Paradiplatys*), but with internal margins armed with one or two teeth, which are sometimes separated by a concavity. Occasionally, a concavity preceding or succeeding the pre-apical tooth is also present but parameres are not divided into two lobes with a cleft (vs cleft in *Nannopygia*). The elongate parameres of the new species, each with an internal tooth but without deep clefts or articulated structures, clearly indicate that the species is a member of *Diplatys*.

Steinmann (1986b) proposed a subgeneric classification system for *Diplatys* (Table 1), mainly based on the relative lengths of virgal regions (paired and unpaird parts), especially those included in the penis lobe. However, some apparently closely related species can be classified into different subgenera according to this system (Gorokhov and Anisyutkin 1994). Therefore, we do not assign *D.sahyadriensis* sp. nov. to any subgenus, as done for some other *Diplatys* species described since Steinmann (1986b) (Table 1).

The male genitalia of *D.sahyadriensis* sp. nov. are unique in the genus in having several elaborations: a well-developed flange on the paired part of virgae, three differently shaped sclerites with serration (or denticulation) on the penis lobe, and a filament-like appendage at each virgal tip. *Diplatysjawalagiriensis*, which has been recorded from Karnataka and Tamil Nadu, also possesses flanged virgae (Kapoor et al. 1971; Srivastava 1988), which indicates a possible relationship with *D.sahyadriensis* sp. nov. According to the descriptions by Kapoor et al. (1971) and Srivastava (1988), however, no conspicuous associated sclerite is present in the penis lobe of this species.

*Diplatyspropinquus* is a Sri Lankan *Diplatys* species possibly close to the new species. According to the descriptions and illustrations by Hincks (1955) and Srivastava (1988), each penis lobe includes three different, serrated or denticulated sclerites, which are very similar to those of *D.sahyadriensis* sp. nov. Little is known of the functions of sclerites observed on the penis lobe of earwigs. However, males of the ovoviviparous spongiphorid *Maravaarachidis* (Yersin, 1860) possess a pair of triangular sclerites (genital hooks) on the penis lobe (Kamimura et al. 2016), and during copulation, the sclerites are firmly pressed against the opening region of the spermatheca, frequently resulting in wounds (Kamimura et al. 2016). At the same time, another spatula-shaped sclerite is shallowly inserted into the spermatheca (female sperm storage organ), supporting the insertion of the narrow (<10 μm in diameter) but highly elongate (ca 20 mm) virga (Kamimura et al. 2016). A similar division of roles among differently shaped accessory sclerites has been reported for another spongiphorid, *Paralabelluladorsalis* (Burmeister, 1838) (Briceño 1997; Kamimura and Ferreira 2017). Different types of denticulated or serrated accessory sclerites have also been reported for several other diplatyids (Hincks 1955; Sakai 1985; Steinmann 1986a; Srivastava 1988). Males of *Diplatysflavicollis* Shiraki, 1907 possess three different types of denticulated sclerites (saber-shaped, rod-shaped, and U-shaped) on each penis lobe (Kamimura 2004). During genital coupling, two lateral pockets in the female genital chamber receive the U- and rod-shaped sclerites, while the saber-shaped sclerite contacts the female subgenital plate (Kamimura 2004). The accessory sclerites of *D.sahyadriensis* sp. nov. may have similar functions in securely holding a female during copulation.

The filament with long spines at each virgal tip represents another characteristic structure of *D.sahyadriensis* sp. nov. In *Diplatys*, the Cameroonian *D.longipennis* Brindle, 1969 also possesses many spines, like barbs, but directly on each tip of the thin, highly elongate paired part of the virgae (Brindle 1969). These barb-like structures may be for the removal of rival sperm from female sperm storage organ(s), as known in several insect groups (Waage 1979). Males of the anisolabidid, *Euborelliaplebeja* (Dohrn, 1863) also use their highly elongate virga, which is usually longer than the entire body, for removing rival sperm from the tubular spermatheca of mates (Kamimura 2000). However, in this species, a recurved flange at the virgal tip is considered responsible for sperm removal (Kamimura 2000). Although males of most earwig species directly insert a virga into the female spermatheca for transferring sperm during copulation (Kamimura 2014; Kamimura et al. 2019), *D.flavicollis* is an exception: the virgal tips are much wider than the spermathecal openings and ducts, indicating that physical removal of stored sperm by a virga is not feasible (Kamimura 2004). However, as in males, female genitalia are quite variable among diplatyid species (Popham 1965; Klass 2003). Future studies on the female genital structures and reproductive biology are warranted for *D.sahyadriensis* sp. nov. and other, related species.

Based on temperature and precipitation, India is divided into six regions: highland, humid subtropical, tropical wet and dry, arid, semiarid, and tropical wet zones (Senapati et al. 2013). *Diplatys* is most species rich in the tropical wet and dry zone (Table 1), which occupies a large part of the southern Indian peninsula. The tropical, rainy climate is responsible for the persistent warm or hot temperature, which normally does not fall below 18°C. India hosts two climatic subtypes, the tropical monsoon climate and the tropical wet and dry climate. The most humid is the tropical monsoon climate, also known as tropical wet climate, which extends over a strip of south-western lowlands abutting the Malabar Coast, the Western Ghats, and southern Assam. These regions are characterised by moderate to high year-round temperatures, even in the foothills, and rainfall which is seasonal but heavy and typically more than 2,000 mm per year (Senapati et al. 2013). Most rainfall occurs between May and November, and this moisture is enough to sustain lush forests and other vegetation for rest of the year. The heavy monsoon rains are responsible for the exceptionally higher biodiversity of earwigs.

The new species of earwig, *D.sahyadriensis* sp. nov. described from sugarcane ecosystem in Western Ghats gives insight into the possible substantial diversity of this genera in India. There is a need to study its distribution, status, and role in agricultural and horticultural ecosystems.

## Supplementary Material

XML Treatment for Diplatys sahyadriensis
